# Weight related health status of patients treated by dietitians in primary care practice: first results of a cohort study

**DOI:** 10.1186/1471-2296-15-161

**Published:** 2014-09-25

**Authors:** Elisabeth Govers, Jacob C Seidell, Marjolein Visser, Ingeborg A Brouwer

**Affiliations:** Department of Health Sciences and the EMGO Institute for Health and Care Research, Faculty of Earth and Life Sciences, VU University Amsterdam, De Boelelaan 1085, 1081 HV Amsterdam, The Netherlands; Department of Epidemiology and Biostatistics, EMGO Institute for Health and Care Research, VU University Medical Center, Amsterdam, The Netherlands; Amstelring Foundation for Primary Care, Amstelveen, The Netherlands

**Keywords:** Dietitian, Weight related health risk level, Patient characteristics, Obesity, Primary care

## Abstract

**Background:**

Overweight and obesity are common in the Netherlands: in 2006 51% of adult men and 42% of adult women were overweight; 10% of men and 12% of women were obese. Patients with overweight or obesity in the Netherlands are often referred to dietitians in primary care for weight loss treatment. We followed a prospective observational cohort to study the effectiveness of this treatment and present the baseline results in this article.

**Methods:**

We invited dietitians throughout the country, who completed at baseline a questionnaire for each patient including weight, stature, waist circumference, age, gender, morbidities, medication, education level, ethnicity, referral, treatment expectations, history of previous weight loss attempts, and exercise.

**Results:**

At baseline data from 1546 patients were obtained from 158 dietitians working in 26 practices. The majority (73%) of patients were obese (BMI ≥ 30 kg/m^2^); and 10% had a BMI of 40 kg/m^2^ or more. The majority of patients (94%) had a high to extremely high weight related health risk (WRHR): (BMI 25–30 kg/m^2^ with comorbidities, or BMI 30–35 kg/m^2^ without comorbidities, up to BMI ≥35 with comorbidities and BMI ≥40 with or without comorbidities). More than half (57%) had comorbidities and a long history of weight loss attempts. An extremely high WRHR was seen in 24.5% of the sample. Patients with very high to extremely high WRHR often had type 2 diabetes mellitus; hypertension; dyslipidaemia; osteo arthritis; and sleep apnoea. Patients of middle and old age had a higher risk for very high and extremely high WRHR. Those with other comorbidities and those who asked for referral themselves had a lower risk.

**Conclusion:**

The study was effective in recruiting dietitians to participate. The sample is representative for dietitians working in primary care. The majority of patients (94%) had a high to extremely high weight related health risk (WRHR).

## Background

In the Netherlands the prevalence of overweight and obesity has risen to 47.2% overweight and 11.8% obese citizens in 2011, in the population from age 15 and up [1]. These figures are based on self-reported weights and therefore likely to be underestimated as both men and women have been shown to under estimate their weight by 26% and 30% [2]. Patients with overweight and obesity - with and without comorbidities - are often referred to dietitians in primary care or visit the dietitian on their own initiative [3, 4]. Dietitians work in primary care practices at the same location as the general physician, or have their own practice place close to the local health facilities. They may work solitarily or be part of a larger organisation that supplies services to a number of practices. They are paid by the health insurance of their patients for a limited number of hours (minimum three, maximum six hours), dependent on the patients’ personal insurance policy.

A recent economic report showed the benefits of treatment by dietitians of patients with multiple weight related chronic conditions in the Netherlands [5]. Several studies have been published in the US and the United Kingdom showing effects of weight loss treatment by dietitians [6–11]. To get an insight in dietary treatment of these patients we designed a prospective observational cohort study in which we included a large number of patients from more than twenty practices of dietitians in primary care across the country. For the complete study we phrased the research question as: “What are the determinants of weight loss and what are the success rates in patients treated by dietitians in primary care?” In this article we describe the characteristics of the population.

## Methods

We designed the study as a prospective observational cohort study in which we followed patients who are treated in dietary practices for weight loss and weight maintenance for two years. We developed questionnaires, based on a survey in 2005, in which methods of weight management of 36 practices of dietitians in primary health care were evaluated, and which were representative for methods commonly used by dietitians. The intervention period ran up to one year; after one year a combination of intervention and weight maintenance took place and the second year long-term was weight maintenance only.

### Recruitment of dietitians

**Figure 1:**
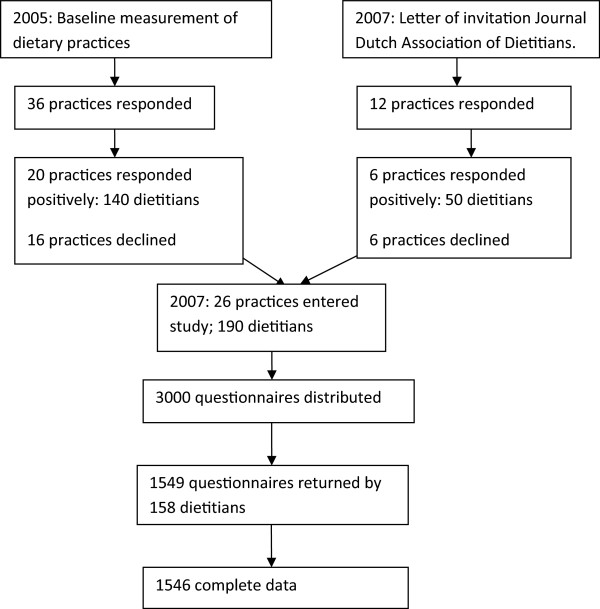
**Study design.**

We approached the 36 practices again and asked dietitians to participate in the current study. As a result 140 dietitians from 20 practices responded positively. Furthermore we added a letter to the Journal of the Dutch Association of Dietitians (2000 copies; 90% of registered dietitians have a subscription to this journal) to invite dietitians in primary health care to volunteer for this study. As a result 50 dietitians from another 12 practices responded positively. Dietitians from six practices decided not to participate because of time constraints. In total 190 dietitians from 26 dietetic practices responded positively. One of the authors (EG) went to each practice to explain the purpose of the study and to train the dietitians on how to collect the data in a standardized way. In addition, the Dutch guideline on obesity treatment was carefully explained.Eight dietitians coordinated the study in their team without treating patients themselves. Finally, of the initial 190, 156 dietitians from 26 practices recruited patients in the study; 26 dietitians decided not to participate in the study (Figure 1) for the following reasons: a too high work load, pregnancy, or seeing too few patients because of management tasks. In 21 of the practices a team of dietitians was involved; five practices consisted of only one dietitian.

### Recruitment of patients

The period of recruitment was from March through July 2007. The dietitians themselves recruited new patients with and without comorbidities who visited on their own initiative or were referred to their practice for weight loss treatment. No specific instructions were provided on how to recruit these patients, other than the recruitment period of three months and the suggestion to recruit consecutive patients. Each patient signed an informed consent form. The dietitians filled out a questionnaire for each included patient that visited their practice for weight loss treatment at the first consultation. The general practitioner of each patient was informed by a letter that was added to the report the dietitian sent to the general practitioner after the first consultation.

Inclusion criteria were: all patients aged older than eighteen years who visited the dietitian for treatment for overweight or obesity with or without morbidities. No maximum age was set and the only exclusion criterion was pregnancy at the start of the treatment. We asked each dietitian to include at least ten patients. Approval for the study was obtained from the Medical Ethical Committee of VU University Medical Centre, Amsterdam, the Netherlands.

### Study sample

We distributed 3000 patient baseline questionnaires to 190 dietitians to be completed. Questionnaires were based on the outcomes of the expert meeting, and on a pilot study we carried out. We chose to start an observational study because it was unclear which patients were seen by the dietitian for weight management. To our knowledge no validated questionnaires on referral, treatment expectations, previous weight loss attempts and other treatment outcomes are available. Therefore we developed questionnaires ourselves. We obtained completed questionnaires from baseline data of 154 dietitians (81%). Dietitians completed all forms themselves. We asked them to do so because we wanted as high a response rate as possible with comparable outcomes, e.g. standardised weighing and measuring. We received data on 1546 adults, 464 men (30%) and 1082 women (Table 1).

**Table 1:** **Baseline characteristics of the adult study sample**

	Total sample (%)	Male (%)	Female (%)	Chi square test
				P value
N	1546	464	1082	
Age year; mean ± SD	49 ± 14.7	52 ± 13.7	48 ± 15.0	
19-44 (young)	402 (26.0)	82 (17.6)	320 (29.5)	<0.001
44-64 (middle aged)	749 (48.4)	234 (50.5)	515 (47.6)	
≥ 65 (old)	395 (25.6)	148 (31.9)	247 (22.9)	
BMI kg/m^2^; mean ± SD	33.5 ± 5.4	33.5 ± 5.0	33.5 ± 5.5	
<25	21 (1.8)	7 (1.5)	14 (1.1)	0.52
25-29.9	386 (24.9)	106 (23.0)	280 (26.0)	
30-34.9	616 (39.8)	199 (43.0)	417 (38.7)	
35-39.9	360 (23.3)	106 (23.0)	254 (23.7)	
≥40	157 (10.2)	44 (9.5)	113 (10.5)	
Weight - kg; mean ± SD	97.8 ± 18.5	108.6 ± 18.3	93.1 ± 16.5	
Waist – cm; mean ± SD	109 ± 13.9	116 ± 12.6	106 ± 13.5	
Waist				
Small^1^	20 (1.4)	9 (2.0)	11 (1.1)	0.26
Medium^2^	78 (5.3)	27 (6.0)	51 (5.0)	
Large^3^	1367 (93.3)	413 (92.0)	954 (93.9)	
Education level				
Low	482 (32.7)	110 (24.7)	372 (36.4)	<0.001
Medium	555 (37.7)	164 (37.0)	391 (38.1)	
High	142 (9.7)	54 (12.0)	88 (8.5)	
Very high	292 (19.9)	117 (26.3)	175 (17.0)	
Comorbidities^4^	906 (57.4)	341 (73.5)	565 (52.2)	<0.001
Type 2 diabetes	459 (29.6)	182 (39.1)	277 (25.5)	<0.001
Hypertension	532 (34.4)	208 (44.8)	326 (30.1)	<0.001
Dyslipidemia	388 (25.1)	163 (35.1)	225 (20.8)	<0.001
Arthritis	101 (6.5)	24 (5.2)	77 (7.2)	0.15
Sleep apnea	60 (3.9)	27 (5.8)	33 (3.2)	0.01
Other morbidities	153 (9.9)	33 (7.1)	120 (11.1)	0.02
Ethnicity				
Dutch	1242 (83.1)	399 (88.5)	843 (80.9)	0.03
Other	251 (16.8)	52 (11.5)	199 (19.1)	

N= 100%; age SE mean: 0.37. Missing: age n = 0; BMI n= 7; weight n= 4; waist n= 115; education level n= 75; ethnicity n= 3. ^1^small <94 cm male; <80cm female; ^2^medium 94-102 cm male; 80-88 cm female; ^3^large >102 cm male; >88 cm female. ^4^comorbidities do not add up to 100% because patients have several comorbidities at the same time.

### Measurements

Measurements are shown in Table 2. The questions and response categories at baseline were based on data from an expert meeting, data from a pilot study [12] and on experiences of the dietitians who have treated patients for overweight and obesity. Patients were classified in 5 BMI categories (BMI <25, 25–29.9; 30–34.5; 35–39.9 and ≥40 kg/m^2^ and three categories of waist circumference (<94 or 80 cm; 94–102 or 80–88 cm, and >102 or >88 cm) [13], and in weight related health risk (WRHR): no WRHR (BMI 20–25 kg/m^2^); light WRHR (BMI 25–30 kg/m^2^ with waist <102 cm (male)/<88 cm (female), without comorbidities); high WRHR (BMI 25–30 kg/m^2^ with waist ≥102/88 cm with comorbidities, or BMI 30–35 kg/m^2^ without comorbidities); very high WRHR (BMI 30–35 kg/m^2^ with comorbidities, or BMI 35–40 kg/m^2^ without comorbidities), and extremely high WRHR (BMI 35–40 kg/m^2^ with comorbidities or BMI >40 kg/m^2^, regardless of comorbidities) [14, 15]. The categories no and light WRHR were combined because of small numbers.

**Table 2:** **Measurements**

Phase	Measurement
**Baseline**	
Patient	Date of birth; gender; stature; weight; waist circumference; ethnicity; educational level; diet history; reasons for weight loss; referral; treatment expectations; self-reported morbidities including: type 2 diabetes mellitus; hypertension; dyslipidaemia; osteoarthritis; sleep apnoea; stomach pains; chronically obstructive pulmonary disease (COPD); hypothyroidism; epilepsy; mental retardation; depression, psychiatric illness and cancer [12–14]; medication, smoking habits
Dietitian	Years of experience, number of patients treated per year; skills and training.
Management	Several questions on food habits; type of treatment; exercise (frequency and duration); used folders and other materials; stage of change; patients own estimation of motivation and success.
**Follow-up 6 months**	
Patient	Weight; waist circumference. If working and social situation has changed; if comorbidities have changed; referral to other health workers; smoking habits; what patients do to maintain their weight loss.
Dietitian	Counselling techniques used; judgement of dietitian if and how much the type of treatment, exercise, personal effectiveness, social support, and mental condition have contributed to successful weight loss.
Management	Type of treatment; exercise in frequency and duration; number of consultations; duration of treatment. Which parts of the treatment have been successful: changes in eating behaviour; more exercise; better physical condition; coping with emotions or social environment; improvement of mental wellbeing and/or personal effectiveness? Is treatment continued or has it ended; why treatment has ended; if relapse has occurred.
**Follow-up 12 and 24 months**	
Patient	Weight; waist circumference. If working and social situation has changed; if comorbidities have changed; referral to other health workers; smoking habits; what patients do to maintain their weight loss.
Dietitian	Counselling techniques; judgement of dietitian if and how much the type of treatment, exercise, personal effectiveness, social support, and mental condition have contributed to successful weight loss. If the dietitian has taken up education to improve her skills in counselling
Management	Type of treatment; exercise in frequency and duration; number of consultations; duration of treatment. Which parts of the treatment have been successful: changes in eating behaviour; more exercise; better physical condition; coping with emotions or social environment; improvement of mental wellbeing and/or personal effectiveness? Is treatment continued or has it ended; why treatment has ended; if relapse has occurred

We instructed the dietitians to measure waist circumference with the patient standing up after a normal expiration. The waist was measured mid-way between the top of the hip bone and the lowest rib. The patients were weighed and height was measured by the dietitians in the practice.

We classified type 2 diabetes mellitus, hypertension, dyslipidaemia, sleep apnoea and arthritis as comorbidities [13, 15].

We distinguished two ethnic groups: Dutch and other ethnicities (Moroccan, Turkish, and Surinamese/Antillean and other countries). Education level was split into four levels: low (primary and secondary school), medium (professional training at secondary level), high (high school), very high (higher professional education or university) [16]. The history of previous weight loss attempts was categorized into four levels: no previous weight loss attempts, one, two, and three or more previous attempts. Within the last category was also included the use of medication to achieve weight loss (n = 25). Referral was categorized into five categories: general practitioner, medical specialist, and psychologist, youth care physician and occupational health physician.

### Analysis

We analysed the data stratified by gender using SPSS 19.0 (IBM SPSS, 2012). For Tables 2, 3, 4 and 5 we performed crosstabs with Chi-squared tests. To adjust for multiple testing we considered a more strict P-value (<0.005) as statistically significant. For Tables 6 and 7 we conducted multivariate, multinomial logistic regression analyses to determine whether age, education level, ethnicity, other morbidities (those not included in the assessment of weight related health risk), previous weight loss attempts and referral were associated with weight related health risk as dependent variable. Potential clustering was not adjusted for in the analyses.

**Table 3:** **Comparison of cohort with data of survey**

	National survey 2007	Current study 2007
Number of patients recorded	4634	1546
Number of practices*	22	26
Number of patients overweight or obese	1955 (42.2%)	619 (40.0%)
Number of patients with overweight or obesity and comorbidities	2237 (48.2%)	906 (57.4)
Male	33.2%	30.1%
Female	66.8%	69.9%
Mean age (years)	45.5	49.7
Total number of patients treated for weight	3503 (75.5%)	1525 (97.4%)
Social economic status		
Low	33.7%	32.7%
Medium	41.7%	37.7%
High	21.5%	9.7%
Very high	Not recorded	19.9%

*Practices in national survey were all stand-alone practices. In our study practices were a mix of stand-alone and large practices of more than 5 dietitians.

**Table 4:** Different characteristics * of adults that visit the dietitian for weight management

	Total sample (%)	Male (%)	Female (%)	Chi square test P value
**Previous weight loss attempts**				
No attempt	454 (29.6)	224 (48.4)	230 (21.4)	<0.001
1 time	334 (21.7)	110 (23.8)	224 (20.8)	
2 times	156 (10.1)	48 (10.4)	108 (10.0)	
≥ 3 times^1^	594 (38.6)	81 (17.4)	513 (47.8)	
**Referral**				
General practitioner	686 (44.3)	270 (58.2)	416 (38.4)	<0.001
Patient asked for referral	738 (47.7)	155 (33.3)	583 (53.9)	<0.001
Medical Specialist	110 (7.1)	34 (7.4)	76 (7.0)	0.66
Psychologist	12 (0.8)	1 (0.2)	11 (1.0)	0.10
Occupational health physician	5 (0.3)	4 (0.9)	1 (0.9)	0.01
**Expectations** ^**2**^				
To lose weight	1191 (77.0)	352 (76.0)	839 (77.5)	0.41
Firm guidance	949 (61.4)	251 (53.8)	698 (64.6)	<0.001
Nutritional advice	620 (40.2)	219 (47.4)	401 (37.5)	0.001
A written diet	249 (16.2)	60 (12.9)	189 (17.9)	0.02
To weigh every week	34 (2.3)	7 (1.7)	29 (2.5)	0.22
Help overcome binge eating	120 (7.8)	13 (2.8)	107 (9.8)	<0.001
Help with relapse	206 (13.4)	40 (8.6)	166 (15.3)	<0.001
Recipes/menus	110 (7.1)	26 (5.6)	84 (7.8)	0.13
**Other treatment outcomes** ^**2**^				
To look better	552 (35.7)	109 (23.5)	443 (40.9)	<0.001
Better health	1269 (82.1)	409 (88.3)	860 (79.4)	<0.001
Feel physically fit	636 (41.1)	168 (36.2)	468 (43.2)	0.01
More self confidence	269 (17.4)	31 (6.7)	238 (22.1)	<0.001

*Previous weight loss attempts, initiative for referral, patients’ treatment expectations and other treatment outcomes. ^1^This value includes using medication to lose weight (n=25). ^2^Values do not add up to 100% because patients were allowed to give several answers. Missing: previous weight loss attempts 8; referral 0; expectations 11; other treatment outcomes 7.

**Table 5:** **Personal characteristics, expectations and treatment goals related to weight related health risk in men**

N = 464	No/Light risk (%)^1^	High risk (%)	Very high risk (%)	Extremely high risk (%)	Chi-square test P value
**Total**	23 (5.0)	137 (29.7)	188 (40.8)	113 (24.5)	0.01
**Age**					
Young	9 (11.0)	28 (34.1)	32 (39.0)	13 (15.0)	<0.001
Middle aged	9 (3.9)	63 (26.8)	92 (39.1)	70 (30.2)	
Old	2 (1.4)	48 (32.4)	67 (45.3)	31 (20.9)	
**Education level**					
Low	2 (1.8)	27 (24.5)	47 (42.7)	34 (30.9)	<0.001
Medium	2 (1.2)	51 (31.3)	76 (46.6)	34 (20.9)	
High	5 (9.3)	11 (20.4)	22 (40.7)	16 (29.6)	
Very high	14 (20.2)	40 (34.8)	38 (33.0)	23 (20.0)	
**Ethnicity**					
Dutch	15 (3.8)	119 (30.1)	163 (41.2)	99 (25.0)	0.001
Other ethnicities	7 (13.4)	14 (26.9)	21 (40.4)	10 (19.3)	
**Comorbidities** ^**2**^					
Type 2 diabetes	1 (0.6)	40 (22.1)	86 (47.5)	54 (29.8)	0.01
Hypertension	3 (1.5)	40 (19.5)	89 (34.4)	73 (35.6)	<0.001
Dyslipedemia	3 (1.9)	40 (24.8)	74 (46.0)	44 (27.3)	0.047
Arthritis	0 (0)	2 (8.3)	13 (54.2)	9 (37.5)	0.091
Sleep apnoea	0 (0)	2 (7.4)	11 (40.7)	14 (51.9)	0.005
Other morbidities	4 (12.2)	14 (42.4)	12 (36.4)	3 (9.1)	0.019
**Previous weight loss attempts**					
Never diet	17 (7.7)	74 (33.5)	101 (45.7)	29 (13.1)	<0.001
1 time	1 (0.9 )	34 (30.9)	43 (39.1)	32 (29.1)	
2 times	1 (2.1)	12 (25.0)	20 (41.7)	15 (31.3)	
≥ 3 times	4 (5.0)	17 (21.0)	24 (29.6)	36 (44.4)	
**Referral**					
General Practioner	6 (2.5)	65 (26.6)	108 (44.3)	65 (26.6)	0.74
Patient asked	17 (10.6)	58 (36.0)	48 (29.8)	38 (23.6)	0.67
Medical Specialist	0	10 (29.4)	16 (47.1)	8 (43.5)	0.58
Psychologist	0	0	1 (100.0)	0	-
Occupational health physician	1 (0.25)	0	1 (0.25)	2 (0.50)	-
**Treatment goal** ^**3**^					
To lose weight	17 (4.9)	106 (30.4)	136 (39.0)	90 (25.8)	0.42
Firm guidance	10 (4.0)	67 (26.90)	102 (41.0)	70 (28.1)	0.23
Nutritional advice	10 (4.7)	72 (33.3)	87 (40.3)	47 (21.8)	0.43
A written diet	4 (6.6)	15 (25.0)	25 (41.7)	16 (26.7)	0.86
To weigh every week	0	1 (14.3)	2 (28.6)	4 (57.1)	0.37
Overcome binge eating	2 (15.4)	1 (7.7)	4 (30.8)	6 (46.2)	0.05
Help with relapse	3 (7.5)	6 (15.0)	12 (30.0)	19 (47.5)	0.004
Recepies/menus	2 (8.0)	7 (28.0)	7 (28.0)	9 (36.0)	0.44
**Other treatment outcomes** ^**3**^					
To look better	11 (10.2)	36 (33.6)	35 (32.7)	25 (23.4)	0.01
Better health	17 (4.2)	116 (28.6)	169 (41.7)	103 (25.4)	0.001
Feel fit	8 (4.8)	42(25.1)	64 (38.3)	53 (31.7)	0.09
Self confidence	3 (9.7)	10 (32.3)	5 (16.1)	13 (41.9)	0.01

No health risk: BMI 20–25 kg/m^2^. Light risk: BMI 25–30 kg/m^2^ with waist <102 cm, without comorbidities*. High risk: BMI 25–30 kg/m^2^ with waist ≥102 cm, with comorbidities, and BMI 30–35 kg/m^2^ without comorbidities. Very high risk: BMI 30–35 kg/m^2^ with comorbidities, BMI 35–40 kg/m^2^ without comorbidities. Extremely high risk: BMI 35–40 kg/m^2^ with co morbidities and BMI >40 kg/m^2^, regardless of comorbidities.

^1^patients with BMI < 25 kg/m^2^were included in light risk: n = 4; ^2^ Comorbidities are: type 2 diabetes mellitus, hypertension, dyslipedemia, and arthritis and sleep apnoea. ^3^Values do not add up to 464 because patients were allowed to give more than one answer; percentages add up within their category. Missing: gender 3; age 3; education level 22; ethnicity 16; previous weight loss attempts 4; referral 20.

**Table 6:** **Personal characteristics, expectations and treatment goals related to weight related health risk in women**

N = 1082	No/Light risk (%)^1^	High risk (%)	Very high risk (%)	Extremely high risk (%)	Chi-square test P value
**Total**	64 (5.9)	404 (37.5)	348 (32.3)	261 (24.2)	0.01
**Age**					
Young	38 (12.1)	153 (47.8)	71 (21.7)	59 (18.4)	<0.001
Middle aged	21 (4.3)	188 (36.6)	172 (33.3)	133 (25.8)	
Old	2 (0.8)	65 (26.9)	110 (44.2)	70 (28.1)	
**Education level**					
Low	7 (1.9)	109 (29.6)	145 (39.4)	107 (29.1)	<0.001
Medium	30 (7.7)	158 (40.5)	110 (28.2)	92 (23.6)	
High	7 (7.9)	34 (38.6)	32 (36.4)	15 (17.0)	
Very high	18 (10.3)	87 (49.7)	40 (22.9)	30 (17.1)	
**Ethnicity**					
Dutch	56 (6.7)	310 (37.0)	276 (32.9)	196 (23.4)	0.35
Other ethnicities	6 (3.0)	78 (39.2)	63 (31.6)	52 (26.2)	
**Comorbidities** ^**2**^					
Type 2 diabetes	0	51 (18.58)	116 (42.5)	109 (39.5)	<0.001
Hypertension	0	61 (18.85)	135 (41.7)	128 (39.5)	<0.001
Dyslipedemia	0	70 (31.1)	96 (42.7)	59 (26.2)	<0.001
Arthritis	0 (0)	11 (14.5)	35 (46.1)	30 (39.5)	<0.001
Sleep apnea	1 (2.9)	4 (11.8)	13 (38.2)	16 (47.1)	0.002
Other morbidities	11 (9.1)	60 (49.6)	26 (21.5)	24 (19.8)	0.001
**Previous weight loss attempts**					
Never diet	16 (7.0)	106 (46.3)	71 (31.0)	36 (15.73)	0.002
1 time	12 (5.4 )	87 (39.0)	75 (33.6)	49 (22.0)	
2 times	10 (9.3)	41 (40.2)	31 (29.06)	23 (21.5)	
≥ 3 times	25 (4.9)	165 (32.3)	168 (32.9)	153 (29.9)	
**Referral**					
General practioner	5 (1.3)	118 (31.8)	133 (35.3)	121 (32.1)	<0.001
Patient asked	54 (8.9)	261 (42.9)	181 (29.7)	113 (18.6)	<0.001
Medical specialist	3 (4.6)	20 (27.4)	24 (32.9)	26 (35.6)	0.37
Psychologist	1 (12.5)	3 (37.5)	1 (12.5)	3 (37.5)	0.12
Occupational health physician	0	0	0	0	-
**Treatment goal** ^**3**^					
To lose weight	51 (6.1)	320 (38.2)	270 (32.3)	196 (23.4)	0.24
Firm guidance	41 (5.9)	254 (36.6)	224 (32.2)	177 (25.43)	0.23
Nutritional advice	28 (7.0)	170 (52.9)	121 (30.5)	78 (19.6)	0.05
A written diet	7 (3.7)	69 (36.9)	68 (33.7)	43 (25.7)	0.45
To weigh every week	0	15 (51.9)	9 (33.)	4 (14.8)	0.38
Overcome binge eating	12 (11.2)	34 (31.8)	34 (31.8)	27 (25.2)	0.13
Help with relapse	10 (6.0)	57 (34.5)	50 (30.3)	48 (29.1)	0.55
Recepies/menus	9 (10.8)	29 (34.9)	28 (33.7)	17 (20.5)	0.21
**Other treatment outcomes** ^**3**^					
To look better	41 (9.3)	192 (43.3)	134 (30.2)	76 (17.2)	<0.001
Better health	27 (3.2)	308 (35.9)	295 (34.4)	227 (26.5)	<0.001
Feel fit	219 (4.6)	175 (37.8)	159 (34.3)	108 (23.3)	0.42
Self confidence	28 (11.7)	106 (44.2)	61 (25.4)	45 (18.8)	<0.001

No health risk: BMI 20–25 kg/m^2^. Light risk: BMI 25–30 kg/m^2^ with waist <88 cm, without comorbidities*. High risk: BMI 25–30 kg/m^2^ with waist ≥88 cm, with comorbidities, and BMI 30–35 kg/m^2^ without comorbidities. Very high risk: BMI 30–35 kg/m^2^ with comorbidities, BMI 35–40 kg/m^2^ without comorbidities. Extremely high risk: BMI 35–40 kg/m^2^ with comorbidities and BMI >40 kg/m^2^, regardless of co morbidities.

^1^patients with BMI < 25 kg/m^2^ were included in light risk: n = 14. ^2^Comorbidities are: type 2 diabetes mellitus, hypertension, dyslipedemia, and arthritis and sleep apnoea. ^3^Values do not add up to 1082 because patients were allowed to give more than one answer; percentages add up within their category. Missing: gender 5; age 5; education level 61; ethnicity 45; previous weight loss attempts 12; referral 15.

**Table 7:** **Results from multivariate multinomial regression analysis with weight related health risk as dependent variable; with no to high weight related health risk as reference in men**

Very high weight related health risk	Odds ratio	95% CI
Age		
Young (reference)	1.0	
Middle age	1.73	(0.92-3.25)
Old	1.28	(0.92-1.80)
Education level		
High/very high (reference)	1.0	
Medium	0.87	(0.49-1.51)
Low	0.90	(0.67-1.20)
Ethnicity		
Dutch (reference)	1.0	
Other	0.90	(0.48-1.67)
Other morbidities		
No comorbidities (reference)	1.0	
Other morbidities	0.52	(0.23-1.19)
Previous weight loss attempts		
None (reference)	1.0	
1 or 2 attempts	1.25	(0.73-2.13)
3 or more attempts	1.37	(0.85-2.21)
Referral		
General practitioner (reference)	1.0	
Self-referred	0.60	(0.34-1.08)
Other health professionals	1.04	(0.45-2.39)
**Extremely high weight related health risk**	**Odds ratio**	**95% CI**
Age		
Young (reference)	1.0	
Middle age	3.72	(1.64-8.48)
Old	1.42	(0.91-2.23)
Education level		
High/very high (reference)	1.0	
Medium	1.41	(0.74-2.72)
Low	1.02	(0.72-1.45)
Ethnicity		
Dutch (reference)	1.0	
Other	0.84	(0.40-1.76)
Other morbidities		
No comorbidities (reference)	1.0	
Other morbidities	0.13	(0.03-0.61)
Previous weight loss attempts		
None (reference)	1.0	
1 or 2 attempts	1.23	(0.67-2.26)
3 or more attempts	1.31	(0.75-2.28)
Referral		
General practitioner (reference)	1.0	
Self-referred	0.87	(0.45-1.70)
Other health professionals	0.70	(0.24/2.02)

*includes stomach pains; chronically obstructive pulmonary disease (COPD); hypothyroidism; epilepsy; mental retardation; depression, psychiatric illness and cancer.

## Results

In total 154 dietitians, which meant 24% of all dietitians in the Netherlands working in primary care at the time of our study, working in 26 practices returned 1549 questionnaires, of which 1546 could be used for analysis (Figure 1). Three questionnaires were excluded because birthdates were missing. The practices were distributed evenly across the country. From all 12 provinces in the Netherlands two or three practices participated. The mean number of patients included in the study was 60 per practice (SD ± 10.4). Per dietitian the mean number of recruited patients was 10 (SD ± 7.2). To examine the representativeness of our sample we compared these data to a national survey (Table 3).

We divided the study population into three age groups (19–44; 45–64; and 65 years and older). The numbers in these age groups were 402; 749 and 395 patients respectively. Differences between men and women in level of obesity, age group, waist circumference, education level, co morbidities, and ethnicity were tested using a Chi-square test (Table 1). The sex differences in history of previous weight loss attempts, referral and treatment expectations were also tested with a Chi-square test (Table 4). The associations between weight related health risk levels and categorical variables including age group, education level, ethnicity, number of comorbidities, frequency of previous diet attempts, referral, treatment goals and treatment outcomes other than weight loss, were analysed with a Chi-square test as well (Tables 5 and 6).

Of the sample 25% was overweight; the majority (73%) was obese (BMI ≥ 30 kg/m^2^); and 10% had a BMI of 40 kg/m^2^ or more. Ninety per cent had a large waist circumference (≥88 cm in women; ≥102 cm in men). More than half of the sample had morbidities. Men had significantly more often type 2 diabetes mellitus (P < 0.001), hypertension (P < 0.001), and dyslipidaemia (P < 0.001) than women.

Women reported more often a history of weight loss attempts, asked for referral more often themselves, and also showed a higher prevalence in their treatment expectation to gain more self-confidence (P < 0.001) (Table 4).

The weight related health risk (WRHR) in relation to sex, age, diet history, and referral and patient treatment goals is presented in Tables 5 and 6. The majority of included patients had a high or very high WRHR. An extremely high WRHR was seen in 24.5% of the sample; 5.6% of the population did not have an increased WRHR. A higher WRHR was related to older age; a lower education level; more previous weight loss attempts; hypertension and the treatment expectation to improve one’s health in men. In women a higher WRHR was related to older age; lower education level; all comorbidities; more previous weight loss attempts; referral, and to treatment expectations like losing weight for a better appearance and better health. A low socioeconomic status was seen in 32.7% of patients and a medium status in 37.7%. A high to very high status was seen in 9% and 19.9% respectively.

Multivariate, multinomial logistic regression analyses (Tables 7 and 8) showed whether these associations were still present after adjustment for other variables. Middle age was consistently associated with a higher risk of very high and extremely high WRHR in men and women. In women, old age was also associated with a higher risk of very high and extremely high WRHR. Having other morbidities was associated with a lower risk of extremely high WRHR in men and women, and a lower risk of very high WRHR in women. Patients who asked for referral themselves had a lower risk of very high WRHR in men (odds ratio (OR) 0.60) and both very high and extremely high WRHR in women (OR 0.64 and OR 0.40).

**Table 8:** **Results from multivariate multinomial regression analysis with weight related health risk as dependent variable; with no to high weight related health risk as reference in women**

Very high weight related health risk	Odds ratio	95% CI
Age		
Young (reference)	1.0	
Middle age	2.25	(1.54-3.26)
Old	1.97	(1.58-2.47)
Education level		
High/very high (reference)	1.0	
Medium	1.01	(0.70-1.48)
Low	0.96	(0.79-1.16)
Ethnicity		
Dutch (reference)	1.0	
Other	0.89	(0.60-1.33)
Other morbidities		
No comorbidities (reference)	1.0	
Other morbidities	0.47	(0.28-0.78)
Previous weight loss attempts		
None (reference)	1.0	
1 or 2 attempts	0.98	(0.70-1.36)
3 or more attempts	1.15	(0.84-1.57)
Referral		
General practitioner (reference)	1.0	
Self-referred	0.65	(0.47-0.90)
Other health professionals	1.17	(0.65-2.11)
**Extremely high weight related health risk**	**Odds ratio**	**95% CI**
Age		
Young (reference)	1.0	
Middle age	1.79	(1.20-2.67)
Old	1.55	(1.22-1.98)
Education level		
High/very high (reference)	1.0	
Medium	1.16	(0.77-1.77)
Low	1.08	(0.87-1.33)
Ethnicity		
Dutch (reference)	1.0	
Other	0.87	(0.56-1.34)
Other morbidities		
No comorbidities (reference)	1.0	
Other morbidities	0.52	(0.39-0.90)
Previous weight loss attempts		
None (reference)	1.0	
1 or 2 attempts	0.85	(0.59-1.22)
3 or more attempts	1.03	(0.74-1.45)
Referral		
General practitioner (reference)	1.0	
Self-referred	0.40	(0.48-0.56)
Other health professionals	1.36	(0.75-2.47)

*includes stomach pains; chronically obstructive pulmonary disease (COPD); hypothyroidism; epilepsy; mental retardation; depression, psychiatric illness and cancer.

## Discussion

The study was effective in recruiting dietitians to participate. The sample is representative for dietitians working in primary care, as well as for patients visiting the dietitian in primary care, compared to a national survey (Table 3). The majority of patients were female, which is in line with the national survey and other patient populations [6, 7], but higher than the samples of several other studies [17, 18]. The men in our study had more comorbidities (73.5%) compared to women (52.2%), but in women weight related health risk was stronger related to comorbidities . This is not consistent with for example the large cohort of Booth et al., who found that comorbidities in men and women were quite similar [19].

In both men and women a high to very high education level was weakly related to a lower WRHR. The high number of patients with a high to very high education level in our cohort is in line with the recent development that obesity is now spread across all SES levels [20]. Our results corroborate that obese patients have a complex profile. In these patients weight management includes dietary treatment of the comorbidities at the same time, thus complicating the treatment. Because of these complexities, weight management should be adjusted to the individual and carried out by dietitians, who have a thorough training in nutrition, dietetics and behaviour, and who can adjust the treatment to the needs, possibilities and expectations of the individual patient to promote lasting weight loss [5]. RD-led dietary instruction in the areas of energy restriction, dietary change, exercise, and behaviour modification was proven more beneficial than frequent weigh-in visits without the RD present in promoting weight loss [9]. A good patient-provider relationship has increasingly been recognized as a critical factor in patient’s treatment adherence [21]. This is in line with the fact that dietitians view themselves as potential leaders in the field of weight management, and see this area as an important part of their role. Registered dietitians have extensive training and hold views that are current, and regularly employ many of the elements of known best practice in management [22]. The management of severely obese patients in primary care may also lead to successful weight loss in patients with morbid obesity [23]. The Dutch criteria for bariatric surgery are similar to the US guidelines: these patients are treated conservatively with diet and lifestyle for one year and are undergo surgery if conservative treatment does not lead to success [24]. Time trends show that the number of patients with a high to extremely high weight related health risk will increase in the near future [25, 26] and dietitians need to be prepared to meet this challenge.

### Main strengths and weaknesses

Our sample consists of a representative sample of 24% of all dietitians working in primary care at the time of inclusion who have voluntarily enrolled in our study. The strengths of this study are the national scale on which it is carried out and the large number of dietitians included, as well as the amount of information about the patients that was collected. These detailed data add to the knowledge of this area of health care.

When we look at geographical dispersal and the balance between larger practices with many dietitians and small private practices the representativeness of our population is sufficient.

A limitation of this study is that we left it up to the dietitians how many patients to include. We have no information of patients that were not included, and who may have been more difficult to treat. A second reason causing selection bias was that some dietitians, who have affinity with treating obesity, have included more patients. Another limitation is that the dietitians and not the patients completed the questionnaires. We chose this method because we wanted to obtain standardised data on medical conditions and anthropometric measurements. On more subjective questions, for example on treatment expectations, our approach may have led to some misinterpretation.

## Conclusion

The study was effective in recruiting dietitians to participate. The sample is representative for dietitians working in primary care. The majority of patients are female despite our efforts to include more men. The majority of patients (94%) have a high to extremely high weight related health risk (WRHR).

## Abbreviations

BMI: Body mass index
WRHR: Weight related health risk
RD: rRegistered dietitian.
